# Supplementary material for: Occupations and Risk of Head and Neck Cancers: A Case–Control Study in Tanzania
Source: Int J Environ Res Public Health. 2025 Oct 29;22(11):1643. doi: 10.3390/ijerph22111643 (PMC12652957; doi:10.3390/ijerph22111643)
Supplement: Supplementary file 1 [file ijerph-22-01643-s001.zip › ijerph-3890908-supplementary.pdf]

Supplementary Material to 'Occupations and risk of head and neck cancers: A case-control study in Tanzania'.

Table S1: Independent Risk Factors for Head & neck cancers based on Multivariate Logistic Regression stratified by duration in agriculture and other work

**Risk factor**	**Cases (n=298) n (%)**	**Control (n=305) n (%)**	**Working duration <10 years** OR (95% CI)	Pvalue	aOR (95% CI)	Pvalue	**Cases (n=298) n (%)**	**Control (n=305) n (%)**	**Working duration >10 years** OR (95% CI)	Pvalue	aOR (95% CI)	Pvalue
***Occupation**												
Non -agriculture	12 (29)	38 (48)	1	1	1		123 (52)	199 (88)	1	1	1	
Agricultural	30 (71)	42 (52)	2.3(1.02-5.04)	0.05	1.6 (0.45-5.49)	<0.48	124 (48)	26 (12)	7.2 (4.46-11.58)	<0.01	5.1 (2.56-9.94)	<0.01
**Age group (years)**												
<40	32 (76)	64 (80)	**1**		**1**		40 (16)	88 (39)	**1**		**1**	
40 - 60	6 (14)	12 (15)	1.0 (0.34-2.91)	1.00	1.4 (0.28-6.76)	0.70	136 (53)	94 (42)	3.2 (2.02-5.03)	<0.01	4.4 (2.25-8.72)	<0.01
>60	4 (11)	4 (5)	2.0 (0.47-8.52)	0.35	0.6 (0.74-4.32)	0.58	80 (31)	43 (19)	4.1 (2.42-6.93)	<0.01	3.1 (1.34-6.70)	<0.01
**Sex**												
Male	18 (43)	27 (34)	**1**		**1**		112 (44)	82 (36)	**1**		**1**	
Female	24 (57)	53 (66)	1.5 (0.68-3.17)	0.32	1.7 (0.53-5.48)	0.34	114 (56)	142 (64)	1.4 (0.94-1.96)	0.10	2.4 (1.35-4.43)	0.01
**Birthplace**												
Urban	12 (29)	45 (56)	**1**		**1**		53 (21)	161 (72)	**1**		**1**	
Rural	30 (71)	35 (44)	3.2 (1.44-7.17)	<0.01	5.4 (1.38-21.06)	0.02	203 (79)	64 (28)	9.6 (6.34-14.65)	<0.01	11.1 (6.14-20.11)	<0.01
**Ever Smoked**												
No	37 (88)	75 (94)	**1**		**1**		177 (69)	190 (84)	**1**		**1**	
Yes	5 (12)	5 (6)	2.0 (0.55-7.44)	0.29	3.5 (0.30-39.98)	0.32	79 (31)	35 (16)	2.4 (1.55-3.79)	<0.01	2.8 (1.35-6.06)	<0.01
**Ever used Alcohol**												
No	31 (74)	61 (76)	**1**		**1**		132 (54)	170 (76)	**1**		**1**	
Yes	11 (26)	19 (24)	1.1 (0.48-2.69)	0.77	1.9 (1.06-3.54)	0.03	119 (46)	55 (24)	2.7 (1.82-3.97)	<0.01	2.4 (1.29-4.43)	<0.01
**Smoked Meat**												
Unknown	12 (28)	13 (16)					87 (34)	35 (16)				
<1 time/week	15 (36)	35 (44)	0.5 (0.17-1.25)	0.13	0.4 (0.19-0.86)	0.02	95 (37)	113 (50)	0.3 (0.17-0.43)	<0.01	0.5 (0.24-1.08)	0.01
1-2 times/week	15 (36	21 (26)	0.8 (0.28-2.16)	0.63	0.6 (0.26-1.38)	0.23	65 (25)	47 (21)	0.6 (0.27-0.77)	0.03	0.6 (0.27-1.47)	0.21
3-5 times/week	0 (0)	11 (14)	-	-	0.2 (0.07-0.72)	0.01	9 (4)	30 (13)	0.1 (0.05-0.28)	<0.01	0.2 (0.06-0.65)	<0.01
**Smoked Fish**												
Unknown	5 (12)	8 (10)	1		1		27 (11)	22 (10)	1		1	
<1 time/week	18 (43)	30 (38)	1.0 (0.27-3.38)	0.95	1.1 (0.45-2.78)	0.23	114 (44)	96 (43)	0.9 (0.52-1.81)	0.92	0.7 (0.31-1.80)	0.51

1-2 times/week	17 (40)	11 (13)	2.5 (0.64-9.54)	0.19	8.1 (1.11-59.97)	0.04	104 (41)	41 (18)	2.1 (1.05-4.03)	0.03	2.3 (0.95-5.50)	0.07
3-5 times/week	2 (5)	31 (39)	0.1 (0.02-0.63)	0.01	0.2 (0.02-2.03)	0.18	11 (4)	66(29)	0.1 (0.06-0.32)	<0.01	0.1 (0.02-0.27)	<0.01
**Chips/ fried potatoes**												
Unknown	13 (31)	10 (12)	1		1		65 (26)	22 (10)	1		1	
<1 time/week	12 (29)	28 (35)	0.3 (0.11-0.96)	0.004	0.2 (0.05-1.22)	0.09	113 (44)	103 (46)	0.4 (0.21-0.65)	<0.01	0.1 (0.01-1.02)	0.05
1-2 times/week	16 (38)	23 (29)	0.5 (0.19-1.52)	0.24	0.4 (0.09-2.18)	0.32	66 (26)	47 (20)	0.5 (0.26-0.88)	0.02	0.2 (0.42-2.49)	0.11
3-5 times/week	1 (2)	19 (24)	0.1 (0.00-0.36)	<0.01	0.1 (0.00-0.93)	0.05	12 (4)	53 (24)	0.1 (0.03-0.17)	<0.01	0.0 (0.0-0.66)	0.03
